# Minimally Invasive Surgery in Acute Bowel Obstruction: Should It Become the Standard of Care? A Prospective, Single Center, Observational Study

**DOI:** 10.3390/jcm13247852

**Published:** 2024-12-23

**Authors:** Hannes Hoi, Martin Grünbart, Michael de Cillia, Robert Uzel, Hannah Hofer, Lisa Schlosser, Peter Tschann, Helmut Weiss, Christof Mittermair

**Affiliations:** 1 Department of General and Visceral Surgery, St. John of God Hospital, Teaching Hospital of the Paracelsus Medical University Salzburg, Kajetanerplatz 1, 5010 Salzburg, Austria; hannes.hoi@bbsalz.at (H.H.);; 2 Department of Internal Medicine, St. John of God Hospital, Teaching Hospital of the Paracelsus Medical University Salzburg, Kajetanerplatz 1, 5010 Salzburg, Austria; 3 Department of Statistics, University of Innsbruck, Universitätsstrasse 15, 6020 Innsbruck, Austria; 4 Department of General, Visceral and Thoracic Surgery, LKH Feldkirch, Teaching Hospital of the Paracelsus Medical University Salzburg, Carinagasse 47, 6807 Feldkirch, Austria

**Keywords:** acute bowel obstruction, minimally invasive surgery, open surgery, laparoscopy

## Abstract

**Background/Objectives**: This study was conducted to determine whether a structured clinical pathway can help to safely implement minimally invasive surgery (MIS) as the standard approach in surgery for acute bowel obstruction (ABO). **Methods**: A prospective analysis was performed on consecutive patients undergoing MIS for ABO at a single center in 2021 and 2022. Prior to the study onset, a structured treatment pathway was defined to apply MIS in all patients. The rate of success in the MIS and patient-, surgeon- and outcome-specific parameters with a focus on complication and conversion rates were analyzed. **Results**: Compared to a historical control group, the conversion rate from MIS to open surgery (OS) nearly doubled from 20.4% to 36.4% (*p* = 0.14). The complication rate in converted patients was 43.8% vs. 7.15% in non-converted patients (*p* < 0.05). **Conclusions**: A structured clinical pathway, including technical standardization and preoperative decisional processes, can be used to implement MIS as a primary surgical treatment in ABO. This is accompanied by high conversion rates and a significant increase in postoperative complications in patients undergoing conversion. Individual decision-making concerning the surgical approach remains paramount to prevent complications and high conversion rates.

## 1. Introduction

Acute bowel obstruction (ABO) is one of the most common occurrences prompting emergency surgery. In contrast to the multitude of elective visceral procedures, the use of minimally invasive surgery (MIS) has not become the standard of care in surgery for ABO. At first glance this seems surprising, bearing in mind the generally accepted advantages of MIS compared to OS, namely a shorter postoperative recovery period, shorter length of stay (LOS), reduced overall morbidity, and better cosmesis [1,2,3]. Moreover, these facts should gain even more attention due to the widespread implementation of current comprehensive strategies such as the “enhanced recovery after surgery” (ERAS) concept [4].

We previously demonstrated that the decision process for or against MIS is not only patient-dependent, but also surgeon-dependent, and was not being implemented even in the hands of surgeons experienced with MIS [5]. This observation led us to scrutinize the former standpoint of surgeons who refused to perform MIS, which we attribute to a lack of sufficient education and training, while also being associated with higher costs than open surgery (OS) and limited exposure of the dissection area, which may increase the risk for bowel perforation [6,7]. The recent MIS rate of 46% calculated for our previous patient cohort is in line with the current literature [8] but would seem to have room for improvement. The use of MIS is of the utmost importance in light of an aging population and overburdened healthcare system if we are to reduce both postoperative morbidity and LOS.

This study is the first to investigate the consequences of implementing MIS in all cases of bowel obstruction under real-world conditions.

Furthermore, it should also assess feasibility and safety by evaluating the risks involved in conversion or reflected in complication rates.

## 2. Materials and Methods

### 2.1. Study Design and Setting

Between 1 January 2021 and 31 December 2022, all patients undergoing emergency abdominal surgery for ABO (study population) at the Department of Surgery of Saint John of God Hospital, Salzburg, Austria, were enrolled in this prospective study. Six experienced senior surgeons who conduct a total of more than 2500 procedures per year, including an average of 350 advanced MIS procedures (colorectal, upper gastrointestinal (GI), liver, and pancreas), performed or proctored all cases to experienced residents (in their 4th to 6th year of residency). Each senior was trained in both MIS and OS and had performed at least 100 advanced MIS procedures (colorectal and upper GI), as well as more than 100 complex OS procedures.

Prior to the study onset, it was the hospital policy that each responsible surgeon had to decide which interventional approach (MIS or OS) to take in patients with ABO. For this prospective study, the following treatment pathway was defined:

1. The official strategy stipulates MIS as the first-choice approach for all patients.
2. The MIS procedural steps for ABO were precisely defined by the following:

Firstly, an initial diagnostic laparoscopy was performed by means of umbilical access either with a 10 mm trocar or a single port system. Secondly, an estimation of the number of adhesions in all four quadrants or the registration of other underlying pathologies (frozen abdomen or previously unknown peritoneal metastatic disease) was carried out by the surgeon in order to decide if immediate strategic conversion would be the better option. Thirdly, additional working trocars were placed on demand.

### 2.2. Selection of Participants

All patients (aged ≥ 16 years) with suspected ABO were seen by an emergency medicine specialist and underwent laboratory blood testing and radiologic imaging (sonography and/or CT scan) to indicate emergency surgery. Patients signed informed consent to participate in this observational study. All patients were seen by an anesthesiologist prior to surgery. Pregnant patients and patients aged ≤15 years are not treated in this hospital, as the hospital policy does not cover the requirements of pediatric and obstetric medical care.

The exclusion criteria for a primary MIS approach were septic or hemodynamically unstable patients, patients undergoing only local redo surgery on the colostomy site, and those with T4 cancer and advanced metastatic disease (these patients served as a comparative group).

### 2.3. Measurements

Procedural data, as well as data from the postoperative course, were documented electronically in an Excel database (Microsoft Excel, Microsoft Corporation, Redmond, WA, USA) and categorized into the following two groups:

1. Patient-specific parameters: age, gender, BMI, ASA score, CRP > 0.5 mg/dL, grade of bowel dilatation (maximum diameter in centimeters), type of surgical approach (MIS versus OS), number of previous abdominal procedures, and reason for ABO (intraoperative findings).
2. Outcome-related parameters: conversion rate from MIS to OS, reason for conversion, and complication rates (complications ≥ Clavien–Dindo 3b).

### 2.4. Outcome Parameters

Conversion and complication rates were defined as primary outcome parameters. The rate of successful MIS was defined as a secondary outcome parameter. Patients who underwent emergency surgery for ABO prior to the study served as the historical control group. In order to assess compliance with the study’s measures, patient- and surgeon-specific parameters were evaluated and compared to the data published previously [5].

### 2.5. Analysis

The statistical analysis was conducted using R, version 4.0.5. All statistical calculations were two-sided, and a significance level of 5% was applied. Group differences were assessed with the Wilcoxon rank sum test for continuous variables and Fisher’s exact test for binary variables. Continuous data are presented as median (25th to 75th percentile) and categorical variables as frequencies (%). The effect size and precision are shown with estimated median differences between groups for continuous data and odds ratios (OR) for binary variables, with 95% confidence intervals (CIs). The sample size calculation was performed by an unpaired t-test with a power of 80% and a significance level of 5%, which revealed a number of 34 patients.

## 3. Results

**Figure 1:**
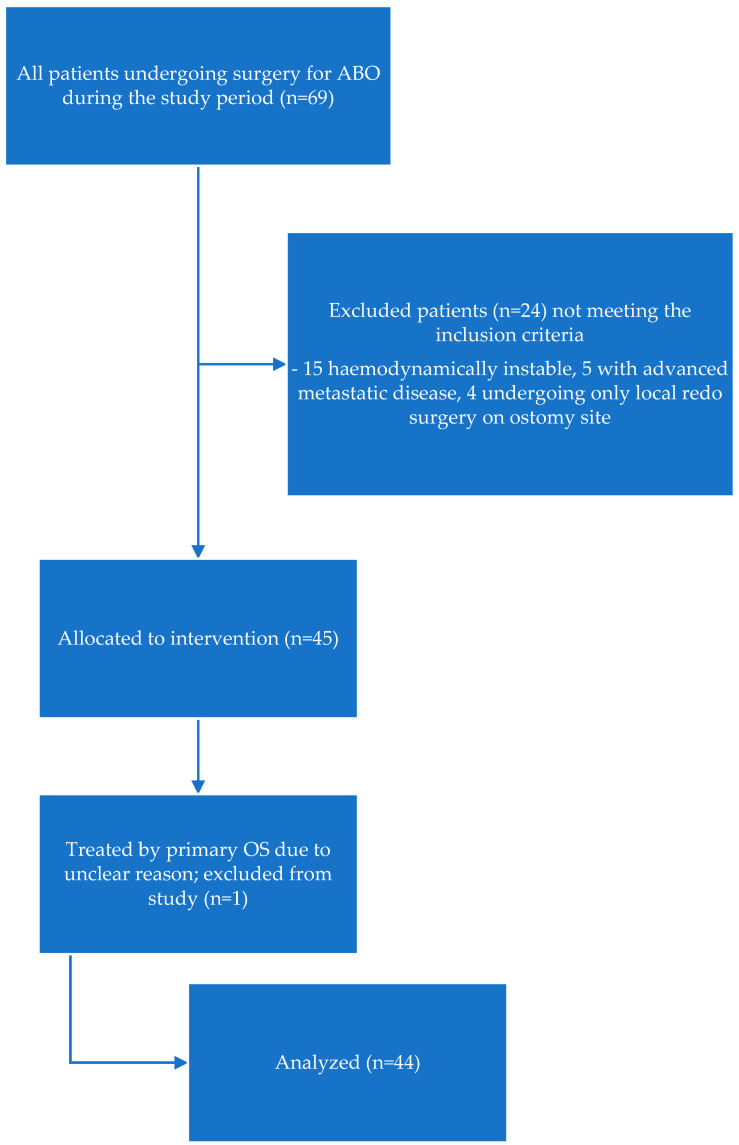
Flow diagram of the inclusion pathway for the study.

A total of 69 patients required surgery for ABO during the study period. A number of patients (24) were excluded from the study because they failed to meet the inclusion criteria. One patient was treated with primary OS for unknown reasons. The inclusion pathway (Figure 1) shows that 44 patients were started on the MIS route. Of those, 28 procedures were completed successfully (63.6%), whereas conversion to OS was necessary in 16 cases (36.4%). Five (31.2%) conversions were performed due to iatrogenic bowel injury, and 11 (68.8%) for strategic reasons (frozen abdomen, severe dense adhesions in all quadrants, or previously unknown peritoneal metastatic disease). The postoperative complication rates, distribution among different groups, and parameters for converted and non-converted patients are given in Table 1.

The 25 patients excluded from the MIS approach due to not meeting the inclusion criteria were all treated by means of OS. These patients had a 40% (10 out of 25) complication rate and showed no significantly longer procedural time than those patients with MIS (73 min versus 62 min, *p* = 0.80).

Intraoperatively, the reasons encountered for ABO comprised adhesions (31 out of 44; 70.5%), tumors (1 out of 44; 2.3%), and others such as incarcerated hernia and inflammatory processes (12 out of 44; 27.3%).

When these study data were compared to our published cohort of patients, who were assigned either to MIS or OS by the surgeon’s free choice and who were undergoing surgery for ABO between 2009–2017, the MIS rate increased from 46.2% (49 out of 106 patients) to 97.8% (44 out of 45 patients), respectively. Although showing no significant difference (*p* = 0.14), the conversion rate to OS nearly doubled from 20.4% (10 out of 49 patients) to 36.4% (16 out of 44 patients) in the historical and study populations, respectively. With respect to the entire cohort of patients undergoing MIS for ABO, this yielded a total of 63.6% successful MIS procedures, which is no longer significantly different from 79.6% of the successful MIS procedures found in the historical population (*p* = 0.14). Regarding the reasons for conversion, we found similar distributions in the study population (5 conversions [31%] due to iatrogenic bowel injury and 11 conversions [69%] due to strategic considerations) and the historical control (2 conversions [20%] due to iatrogenic bowel injuries and 8 conversions [80%] due to strategic considerations).

**Figure 2:**
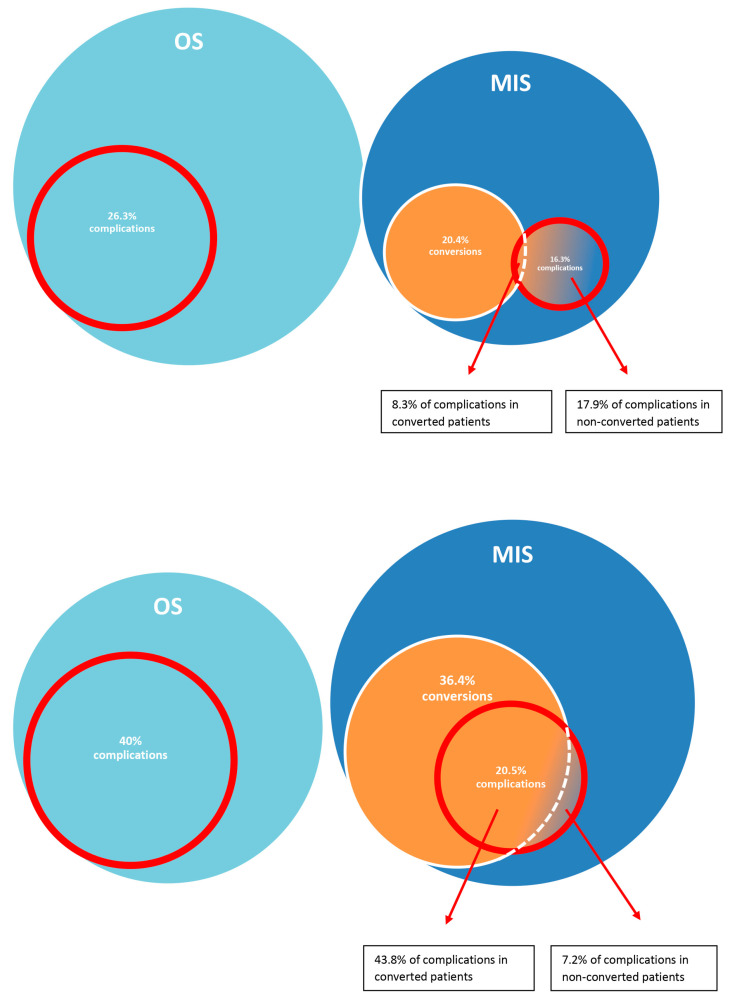
Rates of complications and conversions related to the surgical approach (MIS versus OS) in the control/historical cohort (**upper panel**) and study population (**lower panel**).

Overall complication rates (complications ≥ Clavien–Dindo 3b) were higher in the study patients than in the historical controls: 20.5% (9 out of 44 patients) complications in the study patients versus 16.3% (8 out of 49 patients) in the historical controls (*p* = 0.79). However, patients who were successfully treated by MIS had a lower complication rate of 7.1% in the study group compared to 17.9% in the historical controls. This is paralleled by a higher complication rate in patients with conversions compared to non-converted patients in the study population (43.8% versus 7.1%) but not in the historical controls (10% versus 17.9%); *p* < 0.05. Figure 2 shows a graphic depiction of the conversion and complication rates in the historical and study cohorts.

The types of complications showed a heterogeneous profile in the historical controls as well as in the study population (Table 2).

## 4. Discussion

The results of this prospective study reveal that a targeted package of measures, including technical standardization and the directive to follow a systematic official surgical strategy, can implement MIS as a standard approach of care in surgery for ABO at a center for laparoscopy. However, this is paralleled by the disadvantage of a higher conversion rate and a high complication rate in the converted cohort.

Previous studies confirmed a patient- and surgeon-centered decision process for OS and MIS in patients with ABO [5]. Concerning the general indications for MIS, the current literature promotes this technique even in frail patients, those with previous abdominal surgery, large bowel width, and tumors—although these conditions would warrant caution against using MIS in our historical control cohort [9,10,11,12,13,14,15]. This prospective study was designed to overcome this obvious preconception. A mandatory MIS approach was chosen purposely in all patients except those excluded in advance, which mainly comprised hemodynamically unstable patients and those with advanced metastatic disease, as available data suggest to administer OS in these severely ill patients [16].

A prerequisite for this study was the availability of six expert senior surgeons who either self-performed or supervised all procedures. Assisting surgeons included surgical residents (training years 1–6) and interns. The fact that outcomes did not differ between surgical team constellations underlines the surgical expertise of every individual surgeon. Furthermore, it also reflects the ability to maintain a permanent communication flow between all team members. This is noteworthy, as the current literature reports that communication failures occur in 30% of team exchanges [17].

Regarding the overall percentage of MIS approaches, compared to the historical control group, this study measured significant increases in the percentage of MIS performed for ABO from 46.2% to 63.8% in all patients undergoing surgery for ABO during the study period (including patients not meeting the inclusion criteria for the study). The rather high rate of 46.2% that can be found in the historical controls may be partially due to the fact that our hospital is a center for advanced MIS. However, this finding is in line with published endeavors to reduce surgical trauma when treating ABO: a nationwide retrospective analysis of nearly four million hospital admissions due to ABO in the US between 2001 and 2011 [18] showed an overall MIS rate of 26.5%. This comparably low rate may be due to the early observation time and the large number of participating centers. However, the same study identified a continuous rise in MIS over the years.

Two more recent studies [19,20] revealed MIS rates of about 30% in patients undergoing surgery for ABO. This steady increase in MIS procedures, expressed by a rate of 63.8% MIS in the current study, may reflect the growing awareness, skills, and technical equipment for MIS.

The implementation of MIS as a standard approach in the present study was accompanied by an increase in the conversion rate from 20.4% to 36.4%. It is obvious from the literature that an increase in MIS is paralleled by an increase in conversion to OS. The aforementioned large nationwide US analysis with an MIS rate of only 26.5% showed a relatively low conversion rate of 22.5% [18], whereas the more recent literature [20,21] shows MIS rates of 30% and 32.7% with subsequent conversion rates of 38.6% and 36.7% in ABO. An underlying selection bias in the preoperative decision process is assumed, which over time allocates more critically ill patients to MIS. Interestingly, improvements in surgical skills might influence the higher rates of MIS, but by no means reduced the conversion rates seen throughout the study periods.

**Figure 3:**
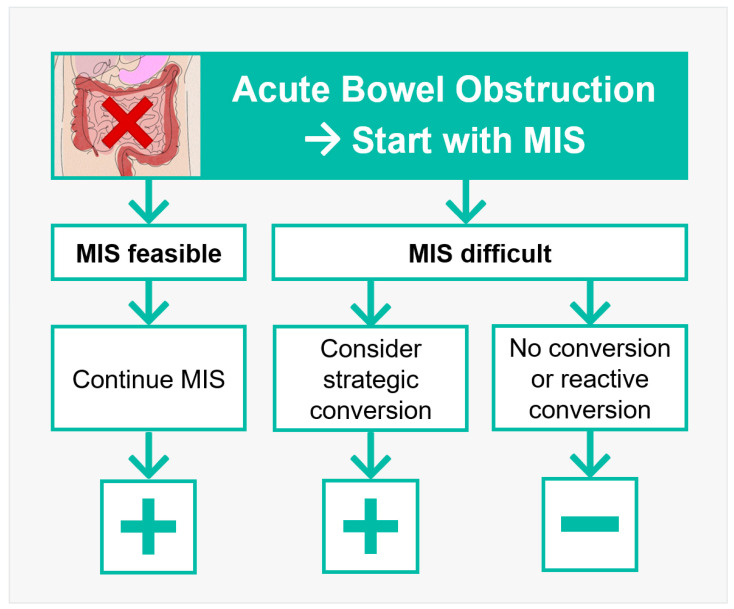
Recommendations for readers to apply MIS in ABO.

An additional factor for a successful increase in MIS rates is a thoughtful decision process in difficult situations. It is known from the literature that reactive conversion is associated with a higher morbidity than strategic conversion [22]. A rate of 31% reactive conversions and 69% strategic conversions was witnessed in this study. Regarding the fact that all patients were treated by means of MIS in this cohort, this number seems acceptable. However, this could be a potential decisive starting point to improve the outcome in MIS for ABO (Figure 3).

Interestingly, no patient-dependent factor (including BMI, age, ASA Score, gender, bowel width, number and type of previous abdominal surgeries, and inflammatory parameters) had an impact on conversion in this study. This is partially in contrast to the findings from Montalti et al. [23], who showed that male patients, patients with previous abdominal surgeries, and those with an ASA score > 2 had an increased risk of undergoing conversion during MIS major hepatectomy. An investigation of 2530 gynecologic laparoscopies revealed a high BMI and a history of previous laparotomy as factors associated with an increased risk for conversion [24]. Possible explanations for the fact that none of these patient-specific parameters were associated with conversion in our results could be a generally higher expertise in the field of MIS nowadays [25], the different complexity of the individual procedures, and the fact that this study was carried out at a MIS-specialized hospital.

Overall, the number of complications (including all patients that were treated with either MIS or OS) increased from 21.7% (23 out of 106 patients) in the historical controls to 27.6% (19 out of 69 patients) during the study period. Patients undergoing primary OS in the historical cohort and patients not meeting the inclusion criteria for the actual study (all OS) showed similar complication rates (14.2% versus 14.5%). However, the study patients showed a higher overall complication rate than the historical controls (20.5% versus 16.3%, *p* = 0.79). This is based on differences between the subgroups and sequelae of the design of the actual study, as a complication rate of 43.8% in the subgroup of patients converted from MIS to OS stands in contrast to the 7.1% found in the non-converted MIS population (*p* < 0.05). Moreover, this is in contrast to a complication rate of 10% in converted MIS versus 17.9% in non-converted MIS patients in the historical controls. Hence, it can be hypothesized that MIS is by far not beneficial for all patients undergoing surgery for ABO. Furthermore, this finding supports the suspicion of a selection bias with a shift of critically ill patients to MIS, which was part of the policy defined in the study design.

These findings strongly advocate for the future implementation of a conversion risk score for patients undergoing surgery for ABO. However, multivariate analysis of our data did not show any significant difference in indication parameters between these groups. A subsequent analysis, including higher patient numbers, is warranted.

### 4.1. Limitations

Improvements in the standardization of clinical and surgical pathways were assured by the single-center character of this study and are not directly applicable to generalization.

None of the preoperative parameters were found to be eligible for patient selection for MIS, as no statistical differences between the converted and the non-converted group could be discerned.

In contrast to the number of authors addressing surgery for ABO, this research includes not only data on patients suffering from adhesive small bowel obstruction but also on any kind of ABO. Although the heterogeneity of underlying diseases could be regarded as a potential limitation, this fact strengthens the significance of this manuscript as it represents everyday clinical practice.

### 4.2. Ideas for Further Research

Even if the prospective character contributes to the study’s statistical power, further randomized controlled trials (RCTs) are required to finally define pathways for the surgical management of ABO. Considering the emergency character of the pathology, the feasibility of those RCTs is limited. In order to provide a “safe study environment” for participants, these should be carried out at MIS-specialized centers. A potential way to guarantee the safety of those trials could be the implementation of initial diagnostic laparoscopy in all patients with ABO, allowing for immediate strategic conversion if needed. The aim of those RCTs should be to define rules for the decision on conversion in order to lower the number and severity of complications.

## 5. Conclusions

In summary, a structured clinical pathway was able to implement MIS as a standard approach in surgery for ABO at a specialized center. However, this was accompanied by the disadvantage of a higher conversion rate. Moreover, converted patients showed a significantly higher rate of complications. Surgeon-specific decisions on the approach of choice should remain mandatory.

## Figures and Tables

**Table 1:** Group comparison of patient- and surgeon-specific parameters, procedural data, and postoperative complication rates in converted and non-converted patients in the study population.

Total (n = 44)	Total (n = 44)	Converted Patients (n = 16)	Non-Converted Patients (n = 28)	Estimate with 95% CI ^a^	*p* Value ^b^
Age	73.05 (61.21–81.53)	67.53 (53.71–78.47)	75.89 (66.74–81.75)	−5.51 (−17.53 to 5.06)	0.2703
**Gender**					
Male	20/44 (45.5%)	5/16 (31.2%)	15/28 (53.6%)	0.4 (0.09 to 1.68)	0.2125
Female	24/44 (54.5%)	11/16 (68.8%)	13/28 (46.4%)	0.4 (0.09 to 1.68)	0.2125
BMI	23.25 (21.38–25.1)	22.95 (22.2–24.38)	23.75 (20.88–25.23)	−0.3 (−2.4 to 2)	0.8452
ASA classification	2 (2–3)	3 (2–3)	2 (2–3)	0 (0 to 1)	0.3426
Number of previous surgeries	2 (1–3)	2 (1–3)	1.5 (1–2.25)	0 (0 to 1)	0.3379
Bowel dilatation (cm)	3.5 (3–3.85)	3.5 (3–3.7)	3.5 (3–4)	0 (−0.5 to 0.3)	0.7582
**Inflammatory parameters (elevated CRP level; yes/no)**					
Yes	22/44 (50%)	6/16 (37.5%)	16/28 (57.1%)	0.46 (0.1 to 1.86)	0.3475
No	22/44 (50%)	10/16 (62.5%)	12/28 (42.9%)	0.46 (0.1 to 1.86)	0.3475
**Type of previous surgery (OS versus MIS)**					
OS	25/30 (83.3%)	10/11 (90.9%)	15/19 (78.9%)	2.59 (0.21 to 144.13)	0.626
MIS	5/30 (16.7%)	1/11 (9.1%)	4/19 (21.1%)	2.59 (0.21 to 144.13)	0.626
**Reason for ABO**					
Adhesive band obstruction	31/44 (70.5%)	12/16 (75%)	19/28 (67.9%)	1.41 (0.3 to 7.71)	0.7385
Tumor	1/44 (2.3%)	0/16 (0%)	1/28 (3.6%)	0 (0 to 68.18)	1
Duration of surgery (min)	61.5 (41.75–116.5)	106.5 (66.75–162.75)	49.5 (38–63.25)	42.67 (16 to 95)	0.0005
**Postoperative complications (yes/no)**					
Yes	9/44 (20.5%)	7/16 (43.8%)	2/28 (7.1%)	9.5 (1.46 to 110.1)	0.0066
No	35/44 (79.5%)	9/16 (56.2%)	26/28 (92.9%)	9.5 (1.46 to 110.1)	0.0066

^a^ Binary data are presented as no./total no. (%), continuous data as medians (25th to 75th percentile). ^b^ Odds ratios for binary variables and estimated median difference for continuous variables.

**Table 2:** Numbers and types of complications in converted MIS, non-converted MIS, and excluded patients (primary OS/control group) in the study population and the historical controls (≥Dindo 3b).

	Anastomotic Leak	Bowel Injury	Persistent Bowel Obstruction	Bleeding	Wound Abscess	Cardio-Pulmonary	Multiorgan Failure	Sepsis	Total
**Numbers and types of complications in the study population:**									
Converted MIS	1	1	1	1	1	1		1	7
Non-converted MIS			1					1	2
Excluded patients (primary OS)	1	1	1	1		3	3		10
**Numbers and types of complications in the historical controls:**									
Converted MIS		1							1
Non-converted MIS		2	4				1		7
OS	2	1	3	2	3		3	1	15

## Data Availability

The datasets generated and analyzed during the current study are not publicly available due to hospital policy, but are available from the corresponding author on reasonable request.

## Abbreviations

MD	Medical Doctor
ABO	acute bowel obstruction
MIS	minimally invasive surgery
OS	open surgery
LOS	length of stay
ERAS	enhanced recovery after surgery
GI	gastrointestinal
ASA	American Society of Anesthesiologists
WA	Washington
USA	United States of America
BMI	Body Mass Index
CRP	C-reactive protein
OR	odds ratio
CI	confidence interval
RCT	randomized controlled trial
EKS	Ethics Committee Salzburg
